# Ghost cells as a two‐phase blood analog fluid: high‐volume and high‐concentration production

**DOI:** 10.1111/aor.14846

**Published:** 2024-08-27

**Authors:** Benjamin J. Schürmann, Pia Creutz, Thomas Schmitz‐Rode, Ulrich Steinseifer, Johanna C. Clauser

**Affiliations:** ^1^ Department of Cardiovascular Engineering, Institute of Applied Medical Engineering University Hospital RWTH Aachen University Aachen Germany; ^2^ Institute of Applied Medical Engineering University Hospital RWTH Aachen University Aachen Germany

**Keywords:** fluorescent hemolysis detection, locally resolved thrombosis detection, particle image velocimetry, resealed ghost cells, translucent two‐phase blood analog fluid

## Abstract

**Background:**

Hemolysis in mechanical circulatory support systems is currently determined quantitatively. To also locally resolve hemolysis, we are developing a fluorescent hemolysis detection method. This requires a translucent two‐phase blood analog fluid combined with particle image velocimetry, an optical flow field measurement. The blood analog fluid is composed of red blood cell surrogates. However, producing surrogates in sufficient volume is a challenge. We therefore present a high‐volume and high‐concentration production for our surrogates: ghost cells, hemoglobin‐depleted erythrocytes.

**Methods:**

In the ghost cell production, the hemoglobin is removed by a repeated controlled osmolar lysis. We have varied the solution mixture, centrifugation time, and centrifugation force in order to increase production efficiency. The production is characterized by measurements of output volume, hematocrit, transparency, and rheology of the blood analog fluid.

**Results:**

The volume of produced ghost cells was significantly increased, and reproducibility was improved. An average production of 389 mL of ghost cells were achieved per day. Those ghost cells diluted in plasma have a rheology similar to blood while being permeable to light.

**Conclusion:**

The volume of ghost cells produced is sufficient for optical measurements as particle image velocimetry in mechanical circulatory support systems. This makes further work on experimental measurements for a locally resolved hemolysis detection possible.

## Graphical abstract

Ghost cell production involves the removal of hemoglobin from porcine red blood cells by repeated controlled osmolar lysis. In our high‐volume, and high‐concentration production, eight 2 L centrifuge beakers (Sorvall BIOS 16, Thermo Electron LED GmbH, Germany) are used to produce 768 mL of ghost cells per production. These ghost cells, diluted in plasma, have a rheology similar to that of blood while being transparent to light. Thus, the ghost cells can be used as a two‐phase blood analog fluid for applications in particle image velocimetry as well as new approaches for local hemolysis and thrombosis detection.

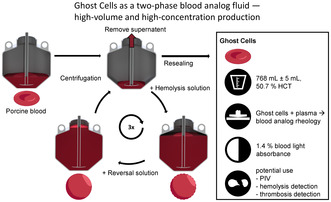

## 1 INTRODUCTION

Hemolysis and thrombosis are the predominant challenges associated with mechanical circulatory support systems (MCSs). The standard hemolysis test according to ASTM1841‐19^1^ compares two MCSs at their operational point in an in vitro blood test. For priming both MCS circuits and a static blood bag a total volume of 900 mL blood at a hematocrit of 35% (315 mL cells) is required. The two MCSs are quantitatively compared over a duration of 6 h based on the free plasma hemoglobin (fHb), which is released from lysed red blood cells (RBCs). However, this hemolysis test lacks information on the location of hemolysis. Therefore, we are developing fluorescent hemolysis detection (FHD) to locally resolve hemolysis in MCSs through an optical measurement. The FHD necessitates a translucent blood analog fluid (BAF), made from resealed ghost cells (GCs) combined with a particle image velocimetry (PIV) setup.^1^, ^2^

The BAF should resemble blood properties as closely as possible and is needed because the light absorption of blood limits optical measurements. As complexity increases, the BAF should mimic the viscosity, the shear‐thinning viscosity, and, ideally, the two‐phase behavior of blood. The most utilized BAF, a mixture of water and glycerol (60:40 wt%), exhibiting a Newtonian viscosity of 3.8 m^2^s^−1^. This viscosity is comparable to that of blood at a shear rate exceeding 1000 s^−1^. To achieve the shear‐thinning viscosity of blood in this mixture, additives such as xanthan gum are employed.^3^, ^4^, ^5^, ^6^, ^7^

In real blood, however, RBCs exhibit two‐phase behavior. To transfer this into BAFs, RBC surrogates are utilized. In their review, Sadek et al.^4^ summarize the requirements for RBC surrogates to be equivalent to RBCs in size, discoidal shape, mechanical properties, and biological functionality. They also state that a high‐throughput production is a big challenge. However, none of the currently used RBC surrogates made of polymers, polysaccharides, or hydrogels meet all of the requirements.^4^, ^8^, ^9^

Therefore, we utilize a two‐phase BAF, which is based on resealed ghost cells (GCs) dispersed in blood plasma. GCs are created by extracting intracellular hemoglobin (Hb) from RBCs. GCs are equivalent to RBCs in size and shape.^2^, ^10^, ^11^ With their intact membrane, they exhibit cellular mechanical properties like the shear‐induced change in shape.^12^, ^13^, ^14^, ^15^ Jansen et al.^16^ demonstrated the usability of GCs as a two‐phase BAF in a small‐scale proof‐of‐principle for PIV.

PIV is a method of measuring the flow field of MCSs optically by tracking the motion of fluorescent particles dissolved in a BAF inside a transparent model of the MCS. The same setup as the PIV combined with the biological functionality of GCs such as possible rupture of the intact membrane makes the FHD possible.

In FHD, the intact membrane of the GCs separates an indicator from its target. Upon hemolysis, when the membrane ruptures, a binding of the indicator to its target is possible, resulting in a fluorescent signal at the location of hemolysis. FHD has been demonstrated as a proof‐of‐principle for chemical hemolysis by Jansen et al.^2^ and needs to be transferred to mechanical hemolysis to be used in MCS testing. Similar to standard hemolysis tests, this requires a sufficient volume of 315 mL of pure GCs, which in turn necessitates a high‐throughput production with a high‐volume and high‐concentration output.

However, this output is limited in the production due to severe cell loss, as described by Ponder et al.^17^ They attributed this cell loss to three possible factors: (1) Inadequate separation of GCs; (2) Shrinkage of GCs; and (3) Non‐conductivity of GCs, which impairs their inductive hematocrit (HCT) measurement.

Schöps et al.^18^ aimed at high‐volume GC production but encountered severe cell loss. This cell loss resulted in only 188 mL of GCs from a starting volume of 800 mL of RBCs. Further, they lacked reproducibility in throughput with a maximal GC concentration of 31.3%.

The goal of this study is establishing a high‐throughput production by overcoming the cell loss through changes in the GCs production. This will enable FHD in MCSs. Additionally, we identified an inconsistency in the previously utilized HCT measurement, indicating that the quantity of GCs produced was overestimated. Consequently, we evaluated different HCT analysis methods.

## 2 METHODS

Our GC production is based on the repeated controlled osmotic lysis technique described by Schöps et al.^19^ We changed the production to increase the throughput of GCs. Furthermore, process reproducibility and GC physiological properties are presented.

### 2.1 Ghost cell production

**FIGURE 1:**
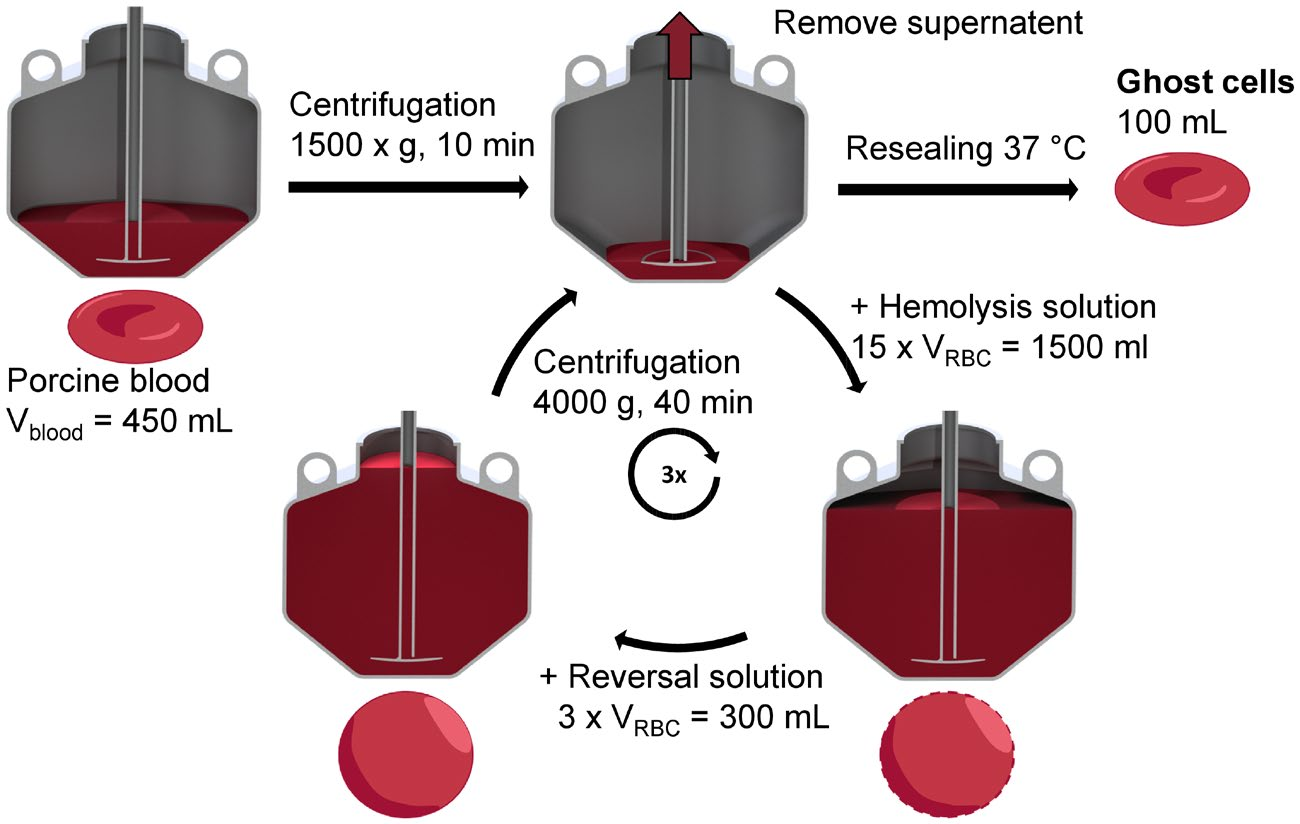
Ghost cell production in one of the eight 2 L beakers of the centrifuge (Sorvall BIOS 16, Thermo Electron LED GmbH, Germany). [Color figure can be viewed at wileyonlinelibrary.com]

Repeated controlled osmotic lysis removes hemoglobin (Hb) of red blood cells. First, a low‐osmolar solution is added to the RBCs followed by a high‐osmolar solution after 5 min. Second, cells are sedimented from the solutions through centrifugation. Third, the supernatant solutions are removed using a vacuum aspirator (Figure 1). The combination of step two and three is referred to as separation. In separation, the Hb is washed away together with the supernatant in successive lysis steps, leaving behind translucent GCs.

A sufficient separation of GCs is vital to avoid cell loss. Separation is depending on the centrifugation time and the sedimentation rate. The sedimentation rate (ϑ) derived from Stokes' law^20^ depends on GCs diameter (*d*), the density difference between the GCs (δp) and the surrounding solution (δL), the viscosity of the surrounding solution (η), and the centrifugation force (g).

$$\vartheta =\frac{d^{2}\left(\mathrm{\delta p}-\mathrm{\delta L}\right)\times g}{18\times \eta }$$

The sedimentation in our production is increased with centrifugal time and force, while the other parameters cannot be changed without altering the controlled osmolar lysis.

Before lysis, RBCs are isolated from porcine blood, obtained by a cut in the jugular vein of pig at a local slaughterhouse. The porcine blood is anticoagulated with a sodium citrate solution (3.13% Eifelfango, Bad Neuenahr‐Ahrweiler, Germany) with a 1:9 ratio and spiked with 0.016 g/L Gentamycin (10 mg/mL Sigma, Taufkirchen, Germany). The blood is filtered to remove bubbles and micro‐thrombi and then centrifuged at 1500 *g* for 15 min (centrifuge: Sorvall BIOS 16, Thermo Electron LED GmbH, Germany). The supernatant of the initial 400 mL of blood per beaker is separated with the vacuum aspirator (Figure 1), leaving a residual RBC pellet of 100 mL. The RBC pellet is loosened using a brush to allow improved mixture in the following lysis.

During lysis, all solutions are pre‐cooled to 4°C, the centrifuge is pre‐cooled to 0°C, and the eight beakers are placed in ice water. In each beaker, the 100 mL RBCs are diluted by adding 1500 mL of low‐osmolar solution with 40 mOsmol/L (1.38 g/L disodium hydrogen phosphate and 1.51 g/L sodium dihydrogen phosphate), followed by 300 mL of high‐osmolar solution with 1660 mOsmol/L (52.6 g/L sodium chloride) with a 5‐min intermediate time. After each dilution, the mixture is shaken. For cell separation, the mixture is centrifuged at 4000 *g* for 40 min at 0°C plus a roll‐out‐time of 20 min due to decreased break settings, in contrast to Schöps et al.^19^ who centrifuged at 3400 *g* for 30 min. To counter cell loss in the first lysis, it is modified in our production by increasing the centrifugation time and residual volume to 60 min and 120 mL, respectively.^19^

After lysis, the GCs in each beaker are washed and resealed with 1000 mL phosphate‐buffered saline at 38°C, followed by separation. Volumes of 180 μL of gentamycin and 160 μL of 50% glucose (B. Braun, Melsungen, Germany) are added to improve the durability of the cells before storing at 4°C for further use. The GCs are characterized by hematocrit, transparency, and rheology and the results are compared with Schöps et al.^18^

### 2.2 Hematocrit

Preliminary results have shown varying HCT for various measurement methods. Therefore, we compare a blood count using the Sysmex XT2000i vet (Sysmex, Germany), a capillary test, and centrifuged 50 mL fractions in Falcons (centrifugation tubes, Fisher Scientific GmbH, Schwerte, Germany) for volume analysis.

During the capillary test, GCs are aspirated into 75 μL glass capillaries, which are then sealed on one side and centrifuged at 21 880 *g* for 10, 40, and 70 min consecutively with measurements in between. HCT is measured by determining the height of the sedimented cell fraction.^21^

Falcon centrifugation (Rotina 420 R, Andreas Hettich GmbH, Tuttlingen, Germany) is performed at 4000 *g* for 60 min at 4°C. The visible amounts of supernatant, GCs, and cellular debris are read off. The Falcon centrifugation is also used to obtain higher concentrated GCs by separating and washing them with PBS, twice.

### 2.3 Transparency

Transparency is assessed as absorbance using triplicate measurements of 200 μL on a 96‐well plate with the Tecan Spark (Tecan Trading AG, Switzerland). The fluids are diluted with PBS to meet the requirement of absorbance measurements to have a resulting value between 0 and 1.

### 2.4 Rheology

The viscosity of GCs and RBCs is measured using a cone‐plate rheometer (MCR502, Anton Paar Germany GmbH, Ostfildern‐Scharnhausen, Germany). Both are adjusted with plasma to HCTs of 30%, 35%, 40%, and 45% and compared to whole blood. RBC rheology is measured at 37°C only, while GCs are also measured at 22°C to simulate laboratory conditions for PIV tests. Also, 10 shear rates ranging from 15 1/s to 1000 1/s are tested, each run for 30 s. The average viscosity of the last 5 s is calculated, and a shear rate of 300 1/s is set between the measurements to neglect transient processes and release possibly formed cell agglomeration.

### 2.5 Statistics

For statistical analysis, the normal distribution is checked with Shapiro–Wilk test and an ANOVA test with the post‐hoc Tukey method is done in RStudio (Posit PBC, Boston, USA).

## 3 RESULTS

### 3.1 Ghost cell production

Our production yields 768.0 mL ± 5.0 mL of GCs in solution partitioned in eight beakers with 96.0 mL ± 2.4 mL each. We characterize the GCs by their hematocrit, transparency, and rheology.

### 3.2 Hematocrit

**FIGURE 2:**
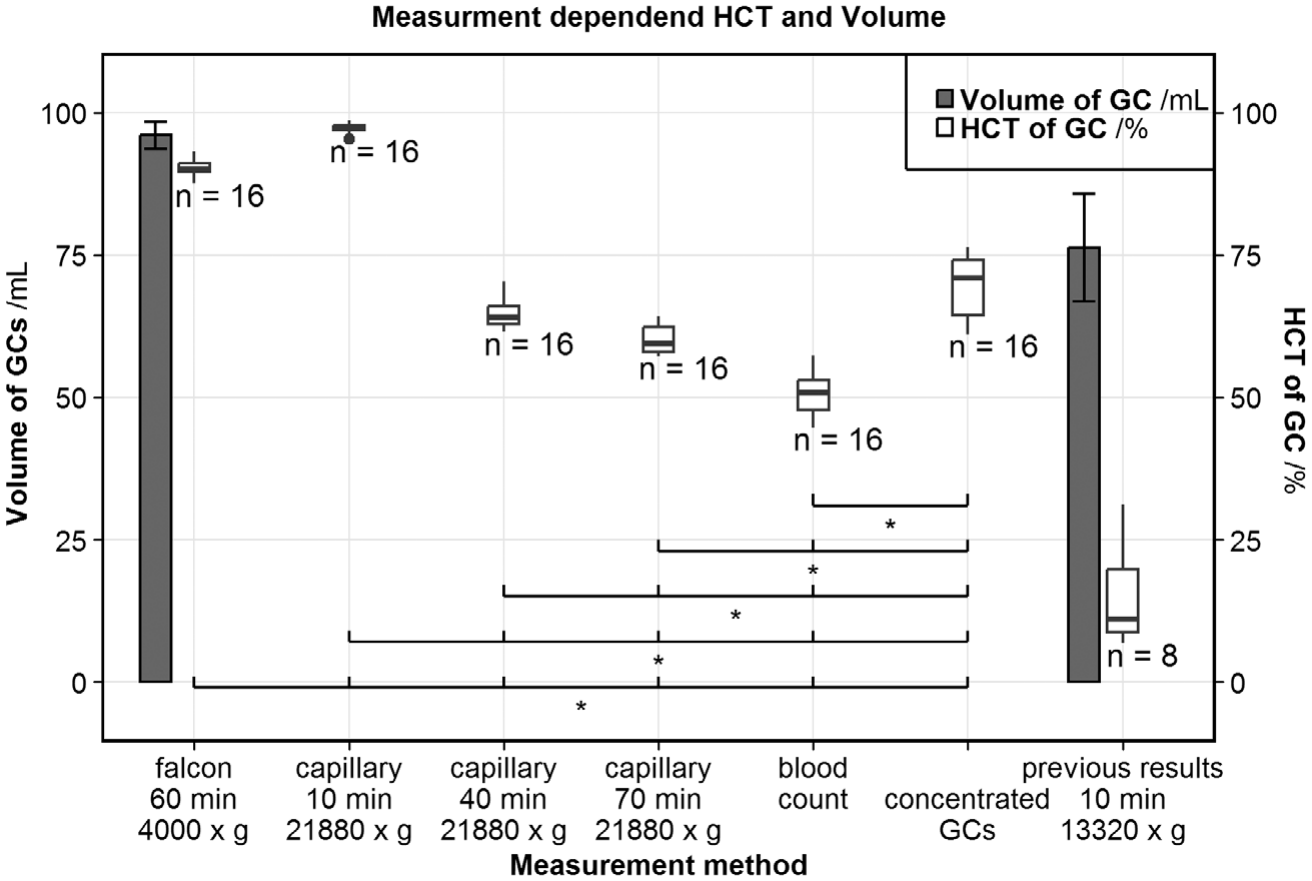
The box plots show the HCT/% separated by measurement methods compared with previous results from Schöps et al.^18^ Normal distribution is checked with the Shapiro–Wilk normality test, the “previous results” showed no normal distribution (*p* = 0.03) and were therefore excluded from the significance analysis with ANOVA and post‐hoc Tukey method (*p* < 0.05). The bar plots show the resulting volume of GC solution/mL per beaker with standard deviation.

For each beaker, we determined the HCT with several measurement methods. Results are shown in Figure 2. Capillary test for 10 min provides a HCT mean of 97.3%. Increased accumulated centrifugation time of 40 and 70 min decreases the HCT to 64.6% and 60.0%, respectively. Falcon centrifugation results in a HCT of 90.3%. In this centrifugation, a pellet of cellular debris 1.1% ± 0.4% sediments below the GCs. The blood count HCT is 50.7%. The high‐concentrated GCs obtained by additional washing had a blood count HCT of 69.2% ± 5.5%.

### 3.3 Transparency

**FIGURE 3:**
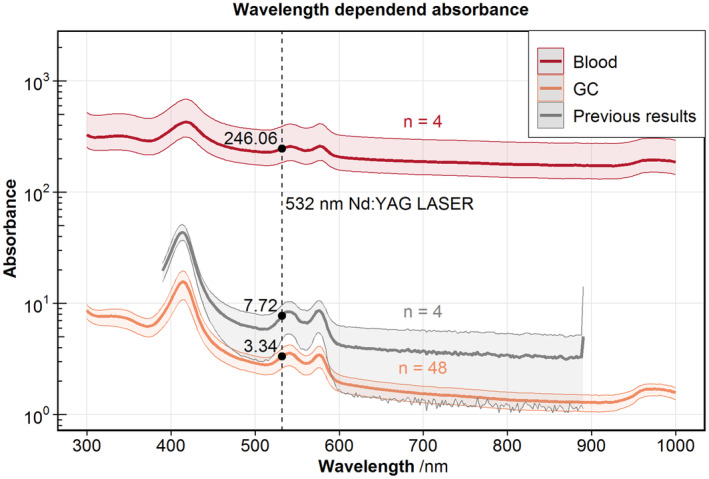
Transparency measurements for GC and whole blood compared with previous results from Schöps et al.^18^ Measurements are normalized to 100% HCT. The ribbon shows the 5% to 95% percentile. [Color figure can be viewed at wileyonlinelibrary.com]

The transparency as absorbance over wavelength is shown in Figure 3. The absorbance at the wavelength of our PIV Nd:YAG laser of 532 nm decreases from 246.06 for whole blood to 3.34 for our GCs. In contrast, the previous production achieved GCs with an absorbance of 7.72 with a larger deviation. All absorbances are normalized to pure GC with 100% HCT.

### 3.4 Rheology

**FIGURE 4:**
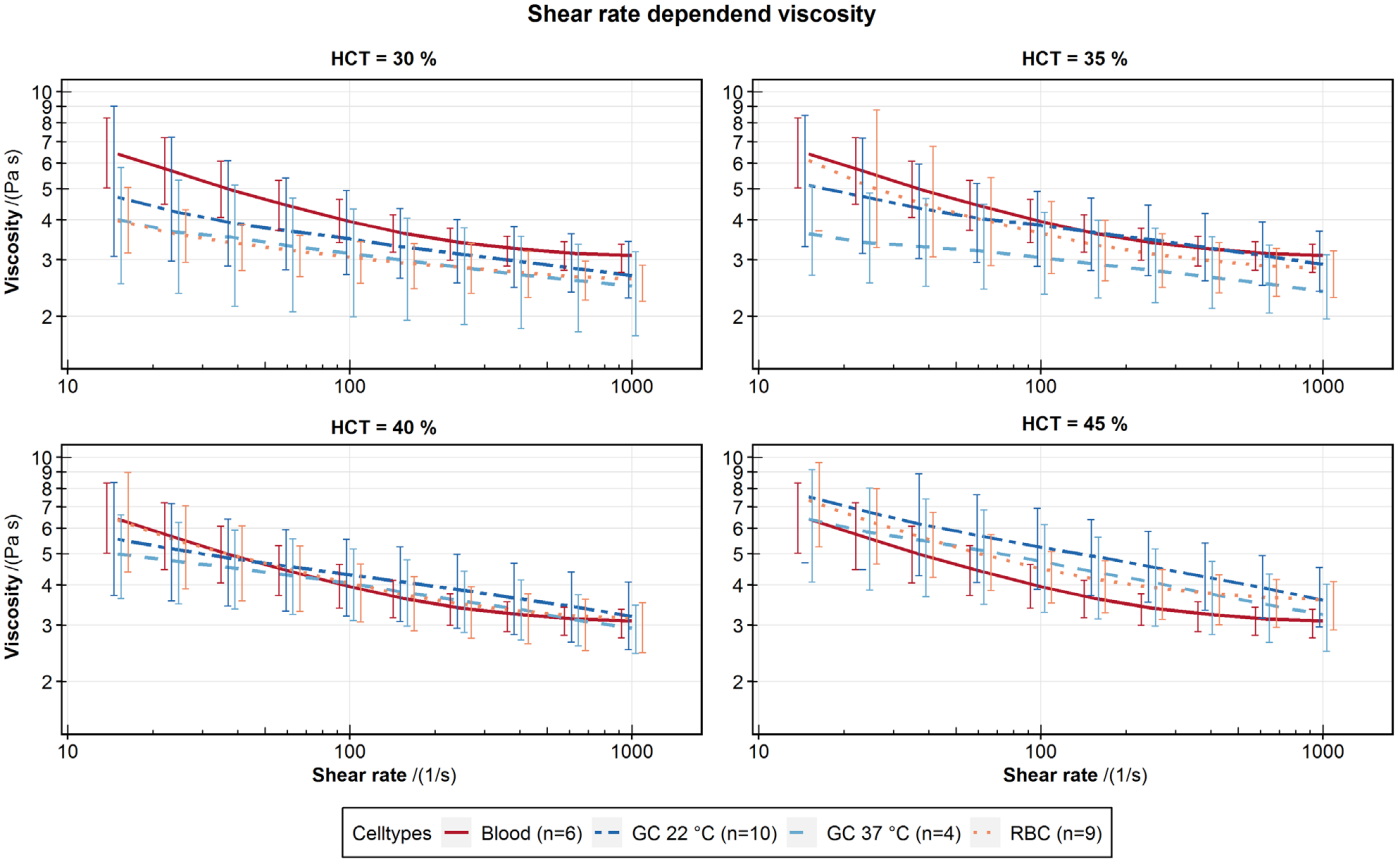
Viscosity of RBCs at 37°C and GCs at 22 and 37°C, adjusted with plasma to set a HCT of 30% to 45%. Comparable measurements of whole blood have a HCT of 40.8% with a standard deviation of 2.5%. [Color figure can be viewed at wileyonlinelibrary.com]

Figure 4 shows the rheology of the concentrated GCs and RBCs with plasma at several HCT as viscosity over shear rate. For both, the viscosity increases with HCT and has a structured viscose behavior. Comparable measurements of whole blood have a HCT of 40.8% ± 2.5%.

## 4 DISCUSSION

### 4.1 Ghost cell production

The objective of this work is to achieve reproducible high‐throughput GC production. To this end, we reduced the cell loss in the production reported in the literature^17^ and increased the output HCT to 50.7% as measured by blood count, resulting in an average throughput of 389 mL of pure GCs per production day. This exceeds our target output volume of 315 mL. Furthermore, the hematocrit of high‐concentrated GCs can be increased to 66.8% ± 5.6% through additional washing in Falcon centrifugation. In contrast, Schöps et al.^19^ produced only 188 mL of GCs with a maximum HCT of 31.3% from the same amount of RBCs.

The reproducibility of the production was achieved by improving solution mixing and by avoiding cell loss. The three possible factors identified by Ponder et al.^17^ that contribute to cell loss and the countermeasures to increase the throughput are discussed in the following.

Ponder's first reason for cell loss is the inadequate separation of GCs. Thus, we increased the separation by increasing the centrifugation time and force (g). Higher forces of up to 20 000 *g* as described in the literature are not possible on our larger centrifuge.^10^ Moreover, the separation is limited by the sedimentation rate, which is dependent on GC diameter (*d*), the density difference between the GCs (δp) and the surrounding solution (δL), and the viscosity (η).

GC diameter can be derived from the mean cell volume (MCV) measured by blood count. The MCV for RBCs is 55.1 fL ± 3.6 fL, while that for GCs is 42.5 fL ± 2.7 fL. For a sphere, this would approximate to a diameter of 4.7 μm for RBCs and 4.3 μm for GCs. The smaller GC diameter has a negative effect on the sedimentation rate by the power of two.

The density of GCs decreases with the loss of Hb, resulting in a much lower density difference between the GCs and the surrounding solution compared to RBCs.^2^ As a result, GCs sediment slower, especially during the first lysis when the solution has a high concentration of Hb released from the cells. This may explain the higher cell loss in the first lysis reported by Schöps et al.^19^ To counter this, we increased the centrifugation time and residual volume for the first lysis. We also reduced the amount of high‐osmolar solution by 41 mL to achieve the targeted osmolarity of 300 mOsmol/L. This reduction of high‐osmolar solution also decreases the density of the surrounding solution, which has a secondary effect on the sedimentation rate.

The viscosity increases with the HCT. Centrifugal cell separation results in an increase in the HCT of GCs in the bottom of the beakers, which in turn leads to a local increase in viscosity and a decrease in the sedimentation rate.^22^ All limiting factors on the sedimentation rate and thereby on the separation are countered within the possibilities of the production to achieve a high throughput of GCs.

Ponder's second reason for cell loss is the shrinkage of GCs and from the measured MCV previously discussed. This leads to a reduction in throughput of 23%. The shrinkage of GCs in the production is a recurring phenomenon and is dependent on the solutions used for the lysis. A further determination of cell size and shape after shrinking is planned for a subsequent study with microscopic cell type evaluation.

Ponder's third reason for cell loss is the impairment of measuring the GCs HCT accurately. We also encountered errors in the HCT measurement. Consequently, we compared several measurement methods to ascertain the total volume and HCT of GC.

### 4.2 HCT measurement

The different measurements of HCT exhibit significant differences between blood count and capillary centrifugation at 10, 40, and 70 min. The blood count HCT measured inductively by the Sysmex XT2000i vet (Sysmex, Germany) may be unable to recognize the GCs due to their lack of hemoglobin. Additionally, the optical measurement detects white blood cells that should not be present after three lyses and washing of the GCs, so some GCs are mistaken for white blood cells. Therefore, the Sysmex may underestimate the cell volume.

Capillary tests rely on centrifugate cell sedimentation as the production, and the height of the sedimented cell is measured. For this height to be equivalent to the HCT, the sedimented cells need to be highly packed. RBCs with their high sedimentation rate and deformability can maximally be packed to approximately 97%.^21^

For GCs, the measured height of packed cells decreases significantly from 10 to 40 min and further to 70 min. While the packing of cells might increase, it is unclear if cells are destroyed in the same process. Therefore, we avoided further elongation of the centrifugation time and expect the maximal packing of GCs somewhere around the HCT of 60.0% at 70 min.

We have not been able to validate the HCT measurements with each other, so it is not possible to determine which measurement is true. To compare the following measurements of transparency and rheology, the HCT is determined based on the blood count.

### 4.3 Transparency

Resealed GCs have an intact membrane but can only be produced with some residual Hb, which leaves some absorption to the cells.^17^ Still, we managed to increase the transparency by reducing the absorption from RBCs to GCs by 98.6%. This increase might be possible due to the increased mixture linked to the reproducibility of the production. It is also possible that with reduced cell loss more GCs with less density are retained. These GCs would then also have less hemoglobin and be more translucent.

### 4.4 Rheology

Two‐phase BAF should have the shear‐thinning viscosity of blood. Therefore, we compared the viscosity of GC mixtures at different HCT to find the most suitable BAF for blood. The viscosity of GC mixtures increases with increasing HCT and exhibits shear thinning, but not as pronounced as whole blood or the RBC plasma mixtures.^22^, ^23^ This difference may relate to the remaining solution in the concentrated GCs. Other possible reasons include differences in agglomeration, shape, or deformation of the GCs. However, the GC mixtures of 35% and 40% HCT at 22°C and the GC mixture of 45% at 37°C show no significant difference to whole blood and are thereby sufficient BAFs.

Other studies used Xanthan to achieve shear‐thinning viscosity in BAF.^3^, ^4^, ^5^, ^6^, ^7^ We have decided against adding Xanthan to adjust the viscosity to maintain a two‐phase fluid with a cellular phase from GCs and a liquid phase, plasma, which has an almost Newtonian viscosity. The resulting two‐phase BAF can be used for PIV measurements. How accurately GCs resemble two‐phase effects of blood like cell‐free layer and Fåhraeus‐Lindqvist effect must be determined in further studies.

### 4.5 Unique application possibilities of GC‐based BAFs

The GCs are adequate RBC surrogates in terms of size, shape, and cellular mechanical properties like shear‐induced change in shape.^2^, ^10^, ^12^, ^13^, ^14^, ^15^ However, the remaining biological function makes them superior to other RBC surrogates, thereby enabling further work on FHD. With the increased production, the FHD previously utilized by Jansen et al.^2^ in small‐scale settings can be applied to operational contexts, such as in a rotary blood pump in MCS.

Moreover, the GC can be utilized for the measurement of live thrombosis. The GCs can be mixed with low‐anticoagulated platelet‐rich plasma. This plasma has the potential to still coagulate within the experimental timeframe and, concurrently, the developing thrombosis can be monitored in real‐time and locally in a MCS with PIV. Clauser et al.^24^ demonstrated such thrombus formation in a plasma‐based BAF as a proof‐of‐principle.

To the best of our knowledge, no other two‐phase BAF has the capability to target this specific usage of RBC surrogates. Given the biocompatibility of the GCs, it is feasible to use natural RBCs at a HCT of 1% in GCs as tracer particles. The behavior of RBCs within a cellular fluid can be analyzed within the translucent GC, which may provide the most realistic representation of real blood. This can be done in two different use‐cases, either by utilizing RBCs as seeding particles in PIV or as cell tracing to observe not only the velocity field but also the deformation patterns of RBCs within a solution of GCs, as previously demonstrated on a smaller scale by Goldsmith et al.^25^

## 5 CONCLUSION

Our presented reproducible high‐throughput production yields unprecedented high volume and high concentration of GCs. The GCs mixed with plasma result in a translucent two‐phase BAF in a sufficient quantity to test entire MCSs. The rheology of this BAF is like the shear‐thinning viscosity of blood, yet it is 98.4% more translucent and thereby allowing optical measurements. The BAF can be utilized not only for PIV to experimentally measure flow fields but also for locally resolved hemolysis and thrombosis measurements, as the remaining biological functionality of GCs allows for such applications.

## AUTHOR CONTRIBUTIONS

Benjamin J. Schürmann: concept, data collection, data analysis, statistics, interpretation, and drafting of article. Pia Creutz: Data collection. Thomas Schmitz‐Rode: Critical revision of article. Ulrich Steinseifer: Critical revision of article. Johanna C. Clauser: concept of article, Critical revision of article.

## CONFLICT OF INTEREST STATEMENT

None.
